# Phosphamide-Based Washing-Durable Flame Retardant for Cotton Fabrics

**DOI:** 10.3390/ma17030630

**Published:** 2024-01-27

**Authors:** Jinhao Li, Guangxian Zhang, Fengxiu Zhang

**Affiliations:** 1 Institute of Bioorganic and Medicinal Chemistry, College of Chemistry and Chemical Engineering, Southwest University, Chongqing 400715, China; 2 State Key Laboratory of Resource Insects, College of Sericulture, Textile and Biomass Sciences, Southwest University, Chongqing 400715, China

**Keywords:** phosphoramidite, flame retardancy, high-efficiency, cotton fabric

## Abstract

A formaldehyde-free reactive flame retardant, an ammonium salt of triethylenetetramine phosphoryl dimethyl ester phosphamide phosphoric acid (ATPEPDPA), was synthesized and characterized using nuclear magnetic resonance (NMR). Fourier transform infrared spectroscopy test (FT-IR), durability test and scanning electron microscopy (SEM) results suggested that ATPEPDPA was successfully grafted on cotton fabrics through a -N-P(=O)-O-C covalent bond. Moreover, the limiting oxygen index (LOI) value of 20 wt% ATPEPDPA-treated cotton was 44.6%, which met stringent washing standard after 50 laundering cycles (LCs). The high washing resistance of the ATPEPDPA-treated cotton was due to the p-π conjugation between the N atom and the P(=O) group in the flame-retardant molecule, which strengthened the stability of the -N-P(=O)-O-C bonds between ATPEPDPA and cellulose, and the -N-P(=O)-(O-CH_3_)_2_ groups in the ATPEPDPA. The cone calorimetric test showed that the treated cotton had excellent flame retardance. In addition, the TG and TG-IR tests suggested that ATPEPDPA performed a condensed flame retardance mechanism. Furthermore, the physical properties and hand feel of the treated cotton were well maintained. These results suggested that introducing -N-P(=O)-(O-CH_3_)_2_ and -N-P(=O)-(ONH_4_)_2_ groups into ATPEPDPA could significantly increase the fire resistance and durability of cotton fabrics.

## 1. Introduction

Cotton is an important cash crop used to produce a variety of products [1,2]. It contains high carbon, oxygen and hydrogen contents, making its fiber flammable [3]. The lack of fire resistance in cotton can limit its applications [4,5]. Thus, improving the fire resistance of cotton products is beneficial to expand their market. Halogen-containing flame retardants are a class of highly efficient flame substances that inhibit combustion by binding reactive radicals (·H and ·OH). However, they have been gradually banned because they produce toxic gases that have carcinogenic effects on organisms [6]. A series of phosphorus-containing flame retardants are applied to cotton fabrics because they are highly efficient and less toxic.

Proban and Pyrovatex CP are the two most widely used phosphorus-containing flame retardants. They can be deposited in cotton by cross-linking them with ammonium or using a cross-linking agent that reacts with cellulose to form C-O-C bonds that inhibit combustion. Textiles prepared with these two flame retardants have excellent flame retardance and durability. However, Proban and Pyrovatex CP involve the use of formaldehyde to form reactive groups during the synthesis. As a result, free formaldehyde will be released during the use of the textiles [7]. Several phosphorus-based flame retardants containing other reactive groups, such as cyanuric chloride groups and vinyl groups, which have been used in cotton textiles [8,9]. However, the flame retardancy and durability of materials can be weak due to poor cross-linking of the flame retardant with the cotton fabric or the groups tend to bulk polymerization.

Biomass has attracted the attention of researchers owing to its advantages of biodegradability, biocompatibility and low toxicity. Common biomasses include wood, vegetables and crops. They contain proteins, phytic acid (PA) and DNA, most of which contain high amounts of phosphorus [10,11,12]. Song et al. finished the fish scale protein and PA onto cotton fabrics using the layer-by-layer technique, and the LOIs of the treated cotton was rose from 17.4 to 31.1% [13]. Alongi et al. coated cotton fabrics with herring DNA to reduce the heat release from the textiles during burning [14]. Biomass flame retardants can enhance the flame retardance of materials; however, the durability of materials needs to be improved after the treatment [15].

Some phosphorus and nitrogen containing ammonium phosphate flame retardants have appeared and are used in cotton fabrics [16,17]. The flame retardants were grafted on cellulose through the dipping–rolling–curing process, and the treated cotton met the AATCC 61-2006 washing standard [16] (washing temperature 49 °C). However, the washing solution contained numerous metal ions (including Ca^2+^ and Mg^2+^). Thus, parts of the C-P(=O)-O-C bond were hydrolyzed, and unreacted -C-P(=O)-ONH_4_ groups combined with the metal ions in tap water to form CaPO_3_-CH_2_- groups. The CaPO_3_-CH_2_- groups generated CaHPO_4_ that cannot efficiently facilitate the char forming, thereby significantly decreasing the flame retardancy. Subsequently, formaldehyde was used in flame retardant synthesis as a reagent to introduce phosphonate groups, which reduced the proportion of ammonium phosphate groups [18,19]. The treated cotton textiles can pass 50 LCs at the AATCC 61-2013 2A [18] standard (50 steel beads, washing at 49 °C). However, cotton fabrics that have been finished are not able to meet the higher standards of washing resistance. Therefore, developing formaldehyde-free and high-efficiency flame retardants is crucial.

In this study, an ammonium salt of triethylenetetramine phosphoryl dimethyl ester phosphamide phosphoric acid (ATPEPDPA) was synthesized and grafted to cotton textiles. During ATPEPDPA synthesis, the -P(=O)-(OCH_3_)_2_ and -P(=O)-(ONH_4_)_2_ were introduced into the triethylenetetramine molecule through a phosphamide reaction, and formaldehyde was not used throughout the synthesis. When -N-P(=O)- covalent bond was formed, the 2p orbitals of the nitrogen atom overlapped and cross-capped with π bond in P=O, thus facilitating the diffusion of the electrons around the N atom to the P atom (i.e., the p-π conjugation). The p-π conjugation decreased polarization between -P(=O) and -(OCH_3_)_2_ in -N-P(=O)-(OCH_3_)_2_ group, which significantly enhanced stability of -P(=O)-(OCH_3_)_2_, making the hydrolysis of covalent bond difficult. After the “dipping-rolling-curing” process, ATPEPDPA was grafted to cotton fibers and formed the -N-P(=O)-O-C bonds. The -N-P(=O)-O-C bond was more stable than the -C-P(=O)-O-C bond, thus preventing the metal ion from binding to -P(=O). Therefore, the ATPEPDPA-treated cotton has an efficient flame retardancy and can be expected to meet the highly restrictive washing standard.

## 2. Experimental

### 2.1. Materials

Methanol (99.5%) and ethanol (99.7%) were supplied by Chuandong Chemical (Group) Co., (Chongqing, China). Triethylenetetramine (95%) was purchased from Zhiyuan Chemical Reagent Co., (Tianjin, China). Phosphorus oxychloride (99.5%) and dicyandiamide (98.5%) were offered through Kelong Chemicals Co., (Chengdu, China). Urea (99%) was produced by Maclean Biochemical Technology Co., (Shanghai, China). Woven cotton fabric (380 g/m^2^, 100%) was purchased from a fabric market (Chongqing, China).

### 2.2. Synthesis of ATPEPDPA

**Figure 1:**
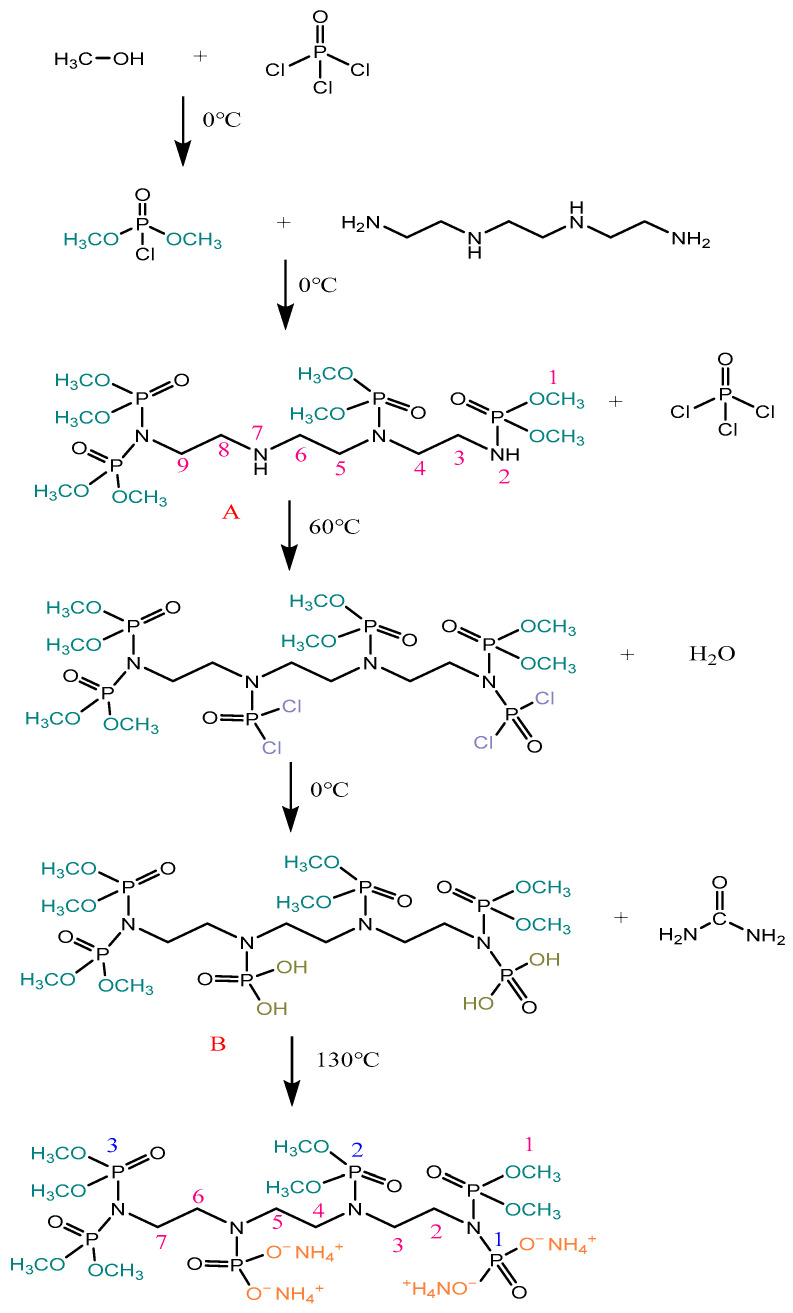
ATPEPDPA synthesis (A is the triethyltetramine phosphoryl tetraphosphate methyl ester, and B is the mixed product).

Methanol (17.690 g, 0.55 mol) was slowly dropped into a triple-necked flask containing phosphorus oxychloride (42.319 g, 0.27 mol) using a constant pressure dropping funnel under an ice bath and stirring conditions, and dimethyl phosphate phosphoryl chloride was obtained. Then, triethylenetetramine (10.091 g, 0.069 mol) was sluggishly added to the dimethyl phosphate phosphoryl chloride to obtain triethyltetramine phosphoryl tetraphosphate methyl ester (A). Afterward, phosphorus oxychloride (21.160 g, 0.14 mol) was added to A at room temperature and placed in an oil bath at 60 °C for 50 min. Then, the distilled water (15.0 mL, 0.84 mol) was added to the intermediate product in an ice bath to obtain the mixed product (B). B was then depressurized at 90 °C to extract the hydrogen chloride gas present in B. Subsequently, the urea (12.5 g, 0.21 mol) was added to the mixture B and heated at 130 °C until the reaction solution pH was 5–6, thus forming a pale-yellow crude product. The crude product was dissolved in distilled water and then purified with anhydrous ethanol to obtain the flame retardant (ATPEPDPA). The hydrogen chloride produced during the reaction was passed through a conduit into a sodium hydroxide solution. Figure 1 shows the synthesis route of flame retardant.

### 2.3. Preparation for ATPEPDPA-Treated Cotton

**Figure 2:**
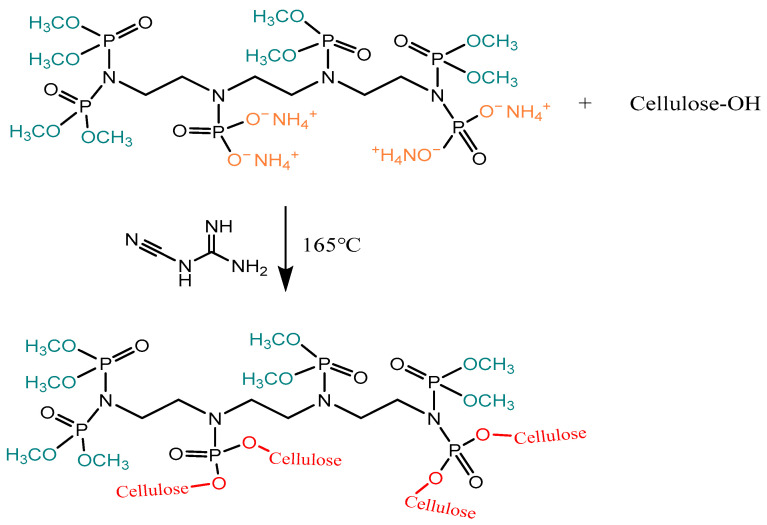
ATPEPDPA reaction with cotton fibers.

ATPEPDPA was dissolved with distilled water and configured with 10, 15 and 20 wt% solutions with 5 wt% of dicyandiamide (as a catalyst) and 15 wt% urea (fiber swelling agent). Raw cotton was mercerized (20 wt% NaOH solution, 13 min) and named control cotton. The control cotton was soaked in an ATPEPDPA solution at 70 °C for 10 min; then, air pressure ginning was performed to evenly distribute the solution on the cotton fabric at 115–125% with liquid rate. Next, the treated cotton was baked at 165 °C for 5 min. After the cotton fabric was washed with distilled water, it was used to evaporate water to a constant weight at 120 °C in a drying oven, and this weight was the real weight after treatment. In the synthesis process, there were several isomerides. One of the possible reactions of ATPEPDPA with cotton fibers is presented in Figure 2.

The weight gain (%) for ATPEPDPA-treated cotton was determined using Formula (1). Table 1 shows the weight gain values of cotton fabrics treated with the flame-retardant concentrations of 10 wt%, 15 wt% and 20 wt%. FRC-X represents the flame retardance cotton treated with X% flame retardant.

*W* = (*W_U_* − *W_F_*)/*W_U_* × 100% (1)

### 2.4. Characterization

The NMR spectrums of samples were analyzed by a nuclear magnetic resonance instrument (AVANCE III, Bruker Biospin Co., Ltd., Fallanden, Switzerland). Deuterium oxide was used to dissolve the flame retardant for the above test.

The functional groups of samples were analyzed using a spectrometer (GX FT-IR, Perkin Elmer Co., Waltham, MA, USA). The samples were swept between 4000 cm^−1^ and 450 cm^−1^ with a precision of 4 cm^−1^.

The cellulose structure of samples was scanned with an X-ray diffractometer (XRD, TD-3500, Dandong Tongda Technology Co., Ltd., Dandong, China). The samples were tested at 20 kV and 20 mA with a measurement range (2θ) of 10–50 ° in steps of 0.02° with Cu Ka radiation (k = 0.154 nm).

The photoelectron spectrum of the samples was tested by an X-ray photoelectron spectrometer (XPS, FEI ESCALAB Xi+, Thermo Fisher Scientific Co., Waltham, MA, USA) and Al was used as an excitation source for X-rays.

The samples’ microstructures were analyzed with a scanning electron microscope instrument (Phenom ProX, Phenom Scientific Co., Ltd., Eindhoven, The Netherlands) with magnifications of 10, 20 and 80 μm. Determination of elemental information for samples was completed using an energy dispersive X-ray spectrometer (EDS, JEOL-6300F, Phenom Scientific Co., Ltd., Eindhoven, The Netherlands).

The durability of the samples was tested with a soaping equipment (JRC-24P, Foshan Jingke Textile Printing and Dyeing Equipment Co., Ltd., Foshan, China) according to the AATCC 61-2013 3A standard: 100 steel balls, 50 mL of 0.15% washing solution and cotton fabrics were soaked into a water washing tank and washed automatically at 71 °C for 45 min. One wash equals five home machine cycles.

The LOIs of samples were obtained with an oxygen index instrument (JL-JF-5, Beijing Beiguangjingyi Instrument Equipment Co., Ltd., Beijing, China). This test followed the ASTM D2863-2000 standard [20], and the size of the sample was 5 cm × 7 cm.

The vertical flaming test of the samples was recorded with flame retardant tester (VFT, YG815B, Nantong Sansi Electromechanical Technology Co., Ltd., Nantong, China). The test complies with the ASTM D6413-99 standard [21], and the sample size was 9 cm × 30 cm.

In different atmospheres (nitrogen, oxygen), samples were characterized for weight loss with increasing temperature using an analytical instrument (Pyris 1, Perkin Elmer Co., USA). The test range was from 40 °C to 700 °C with a 20 °C/min ramp rate. Thermogravimetric-infrared instrument (Nicolet iS10, Thermo Fisher Scientific Co., USA) was used for detection of gaseous species during pyrolysis of cotton fabrics.

The combustion properties of control cotton and ATPEPDPA-treated cotton were tested using a cone calorimeter (6810, Suzhou Vouch Testing Technology Co., Ltd., Suzhou, China) according to the ISO 5660-1 standard [22]. The heat flux was 35 kW/m^2^, and the size of samples was 10 cm × 10 cm. The thickness of samples was 0.60 mm. The relevant data such as heat release rate, total heat release and total smoke release of the samples were recorded.

The samples were performed for whiteness according to the AATCC 110-2000 standard on a spectrophotometer (650, Datacolor Co., Lawrenceville, NJ, USA) with light source D65 and CIE 10° standard viewer.

The samples were obtained for tensile strength on a machine (YM065A, Laizhou Yuanmore Electromechanical Equipment Co., Ltd., Laizhou, China) according to the ASTM D5035-2006 standard [23]. The sample size was 2 cm × 5 cm, and both warp and weft directions were tested. The instrument stretched the sample at a speed of 300 mm/min.

The bending length values of the samples were determined by a fabric stiffness tester (YG(B)022D, Wenzhou Darong Textile Instrument Co., Ltd., Wenzhou, China) in accordance with ASTM D1388-96 (2002) [24]. The sample size was 2.5 cm × 25 cm and was both tested in the warp and weft directions. The test angle was 45° and the sample was moved at a speed of 120 mm/min.

## 3. Results and Discussion

### 3.1. Structural Analysis of Samples

**Figure 3:**
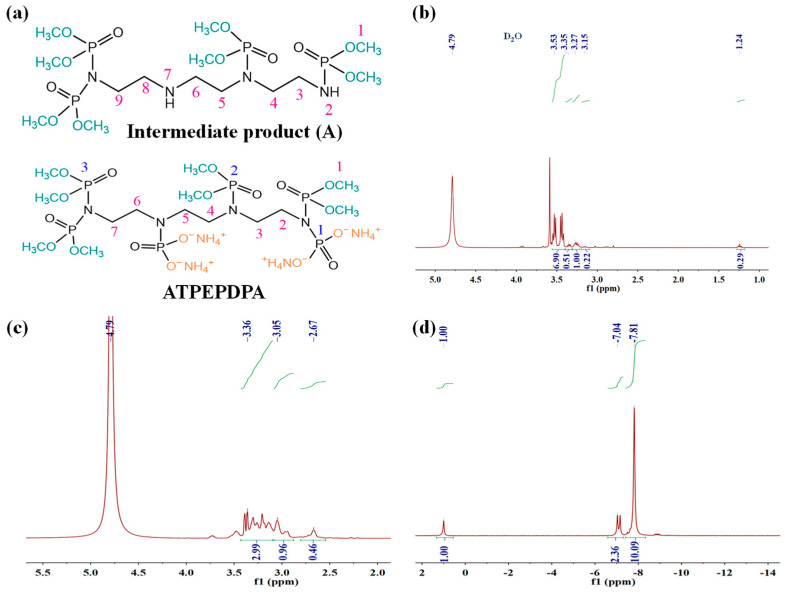
Chemical structure of Intermediate product (A) and ATPEPDPA (**a**); ^1^H NMR spectra of Intermediate product (**b**); ^1^H NMR (**c**) and ^31^P NMR (**d**) spectra of ATPEPDPA.

NMR data of samples are as follows and the spectra are shown in Figure 3. Intermediate product (A): ^1^H NMR (D_2_O; δ (ppm)):4.79 (D_2_O), 3.53 (25H, H_1_, H_2_), 3.35 (4H, H_5_, H_8_), 3.27 (6H, H_3_, H_4_, H_9_), 3.15 (2H, H_6_), 1.24 (1H, H_7_). ATPEPDPA: ^1^H NMR (D_2_O; δ (ppm)):4.79 (D_2_O), 3.36 (24H, H_1_), 3.05 (8H, H_2_, H_3_, H_6_, H_7_), 2.67 (4H, H_4_, H_5_); ^31^P NMR (D_2_O; δ (ppm)):1.00 (P_1_), −7.04 (P_2_), −7.81 (P_3_).

**Figure 4:**
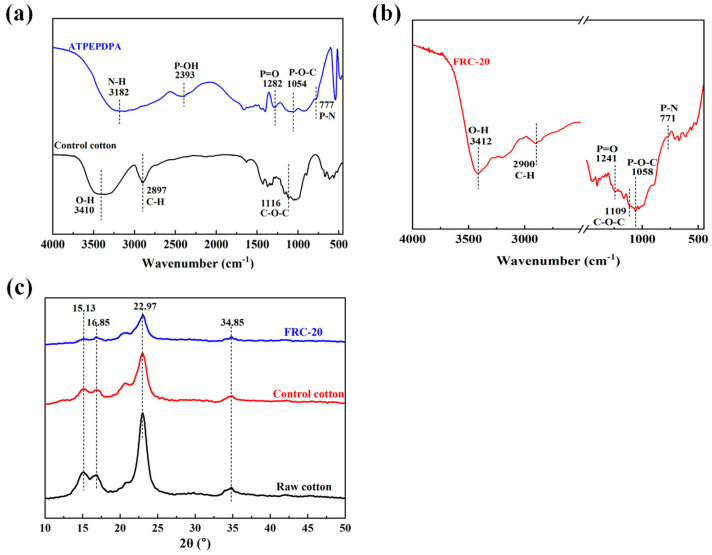
FT-IR patterns for ATPEPDPA (**a**), control cotton (**a**) and FRC-20 (**b**); XRD spectra of FRC-20, control cotton and raw cotton (**c**).

Figure 4a,b shows FT-IR patterns of the ATPEPDPA and cotton before and after ATPEPDPA treatment. Three characteristic peaks at 3410, 2897 and 1116 cm^−1^ were observed in the control cotton, corresponding to O-H, C-H and C-O-C stretching vibrations, respectively [25]. The five peaks at 3182, 2393, 1282, 1054 and 777 cm^−1^ corresponding to N-H, P-OH, P=O, P-O-C and P-N, respectively, were observed in the ATPEPDPA curve, which is consistent with the ATPEPDPA structure [26,27,28]. In the ATPEPDPA-treated cotton (FRC-20) curve, except for characteristic peaks of cotton fibers, three characteristic peaks appeared at 1241, 1058 and 771 cm^−1^, representing P=O, P-O-C and P-N, respectively. Combined with the durability test, this implied that ATPEPDPA grafted with cotton fabric formed -N-P(=O)-O-CH_2_- covalent bonds.

Figure 4c shows the alteration in crystalline shape for cotton fibers by ATPEPPA. The characteristic crests of the raw cotton at 15.13°, 16.85°, 22.97° and 34.85° belonged to cellulose Ⅰ [29]. After the treatment of cotton fibe rs with a high concentration of alkali solution, the peak intensity of the control cotton became lower at these positions. Compared to the control cotton curve, the FRC-20 curve displayed peaks of cellulose Ⅰ, with no significant change observed in the intensity of the peaks, which implied that the effect of ATPEPDPA on cotton fiber structure was relatively weak.

**Figure 5:**
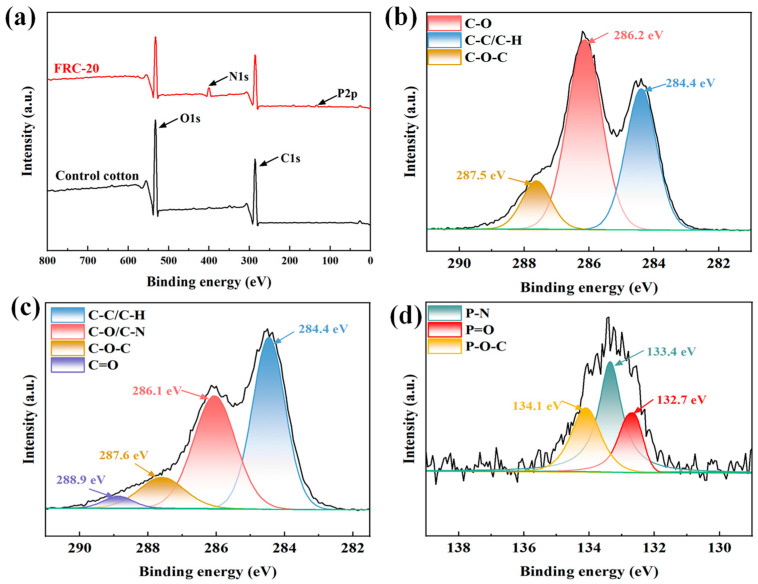
Wide-scan XPS spectra of the FRC-20 and control cotton (**a**); C 1s spectrum of control cotton (**b**); C 1s (**c**) and P 2p (**d**) spectrum of FRC-20.

Figure 5 represents further confirmation of the chemical structures for control cotton and FRC-20 using XPS. The wide-scan XPS spectra showed that FRC-20 contained C, O, P and N elements, whereas the control cotton had only C and O elements. The C 1s spectra of control cotton at 284.4, 286.2 and 287.5 eV corresponded to the C-C/C-H, C-O and C-O-C bonds of cellulose, respectively [30,31,32]. For FRC-20, the peaks at 284.4, 286.1, 287.6 and 288.9 eV corresponded to the C-C/C-H, C-O/C-N, C-O-C and C=O bonds of cellulose and ATPEPDPA, respectively [33]. There are three summits in the P 2p spectrum of FRC-20, located at 132.7, 133.4 and 134.1 eV that corresponded to the P=O, P-N and P-O-C bonds, respectively [34,35]. These results suggested that ATPEPDPA was successfully grafted to cotton fabrics, which was in accordance with the FT-IR findings.

### 3.2. Characterization of Surface Morphology and Element

**Figure 6:**
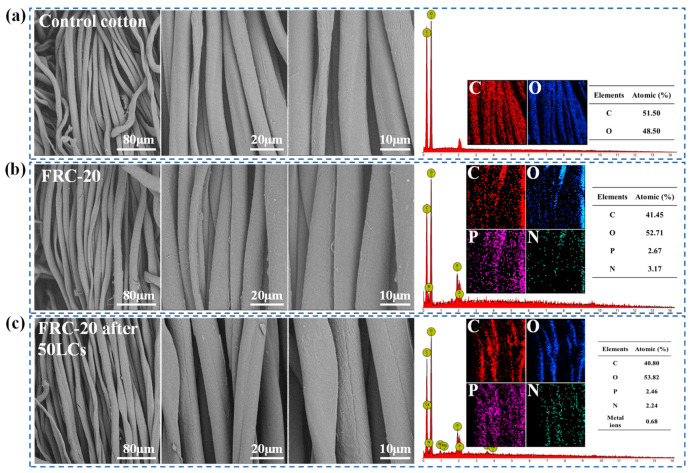
SEM pictures and EDS analysis for control cotton (**a**), FRC-20 (**b**) and FRC-20 after 50 LCs (**c**).

Figure 6 represents the fiber micro-regions of the samples that were characterized using SEM and EDS. After the cotton was treated with alkali solution, the control cotton fibers had a circular and clean surface, and only carbon and oxygen elements were observed. The FRC-20 had a similar surface morphology, but contained an additional 2.67% phosphorus and 3.17% nitrogen elements. The phosphorus and nitrogen contents of FRC-20 after 50 LCs were 2.46 and 2.24%, respectively. Moreover, ATPEPDPA-treated cotton had no noticeable material coating on the surface of the cotton fabric, suggesting that ATPEPDPA entered into the internal cotton fibers rather than being coated on cellulose.

### 3.3. Flame Resistance and Durability of Samples

**Figure 7:**
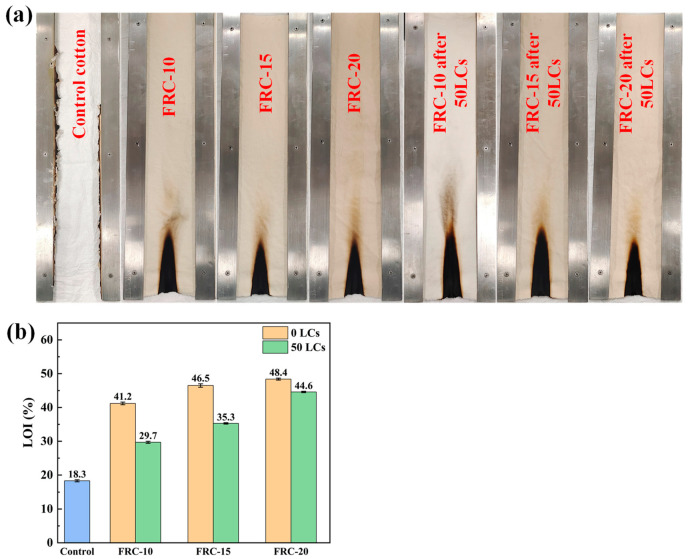
VFT pictures of samples (**a**); LOIs for control cotton and ATPEPDPA finished cotton after different LCs (**b**).

The results for flame resistance of samples are presented in Figure 7 and Table 2. The control cotton was completely burned with a few embers remaining. The structure of ATPEPDPA-treated cotton was intact after burning but turned into a char layer at the cotton part in contact with flame. The higher concentration of ATPEPDPA in the treated cotton shortened the lengths of char, which were 5.7, 5.1 and 4.8 cm. The char length of FRC-20 was 5.2 cm after 50 LCs according to AATCC 61-2013 3A standard, and it can still maintain no after-flame and no after-glow. These showed that ATPEPDPA-treated cotton has good fire resistance.

The LOI values of FRC-10, FRC-15 and FRC-20 were 41.2, 46.5 and 48.4%, respectively, which were significantly higher than control cotton (18.3%). The FRC-10 showed an LOI value of 29.7% after 50 LCs, remaining higher as compared to the flame retardancy standard (26%), which means that ATPEPDPA-treated cotton are considered durable cotton fabrics.

Compared with the small molecule molecular weight flame retardants containing only ammonium phosphonate groups (ammonium pentaerythritol tetraphosphoric acid flame retardant (APTTP)-treated cotton and ammonium phosphate active groups’ flame retardant (AATMPEG)-treated cotton) [36,37], the flame retardants containing phosphonate ester and ammonium phosphonate groups (diethylenetriamine pentamethyl triphosphonate methyl ester ammonium diphosphonate flame retardant (DETA-P)-treated cotton) [18], and the high-molecular-weight flame retardants containing only ammonium phosphate groups (ammonium starch phosphate flame retardant (ASTP)-treated cotton) [38], the ATPEPDPA-treated cotton (FRC-20) met the more stringent washing standards (Table 3). The reason for this phenomenon was the p-π conjunction effect between the N atom and the P=O bond. The effect increased the electron cloud of the P(=O)-O covalent bond; as a result, their polarization decreased and became more stable, and the -N-P(=O)-O-C was not easily hydrolyzed to combine metal ions, such as Ca^2+^. Therefore, the flame retardant in cotton fibers could not be easily washed away and combined with metal ions to lose flame retardancy. In addition, ATPEPDPA was a small-molecule flame retardant, which entered the internal cavity for fibers through dipping and rolling, and a large number of ATPEPDPA molecules grafted with the cotton fiber during curing. In contrast, the high-molecular-weight flame retardants could not enter into the internal cavity of fibers; they only reacted with the hydroxyl groups on the surface of cotton fabrics. Then, more ammonium phosphoric acid groups were observed in flame retardant that did not react with the hydroxyl groups than those in ATPEPDPA; consequently, ammonium phosphoric acid groups combined metal ions during the washing process to form CaPO_3_- groups and decreased flame retardancy. Thus, the ATPEPDPA-treated cotton has excellent durability.

### 3.4. Thermogravimetric Analysis

**Figure 8:**
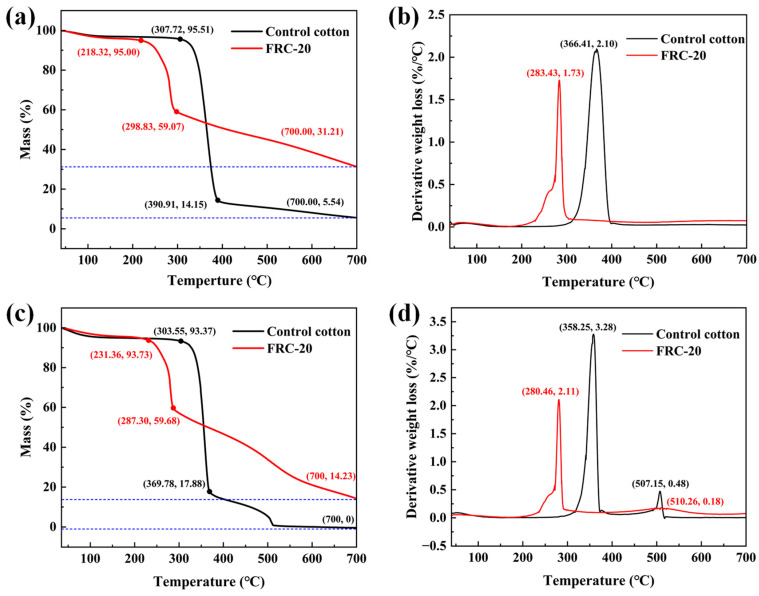
TG and DTG profiles for samples in nitrogen (**a**,**b**) and air (**c**,**d**) atmosphere.

Figure 8 shows the weight loss process of samples with increasing temperature. Under nitrogen atmosphere, the control cotton exhibited a rapid and continuous weight loss with a residue weight of 5.54% remaining at 700 °C. After initial pyrolysis, the cotton fibers were dehydrated and produced a small amount of levoglucose. Then, the production of levoglucose rapidly increased due to the chain reaction, with only a small residue remaining at the end, as shown in two inflection points of the curve (307.72 °C, 390.91 °C). Due to the ATPEPDPA treatment, the cotton fiber advanced at inflection points and maximum weight loss temperature during the heating process (218.32, 298.83 and 283.43 °C), and a high residue was obtained (31.21% vs. 5.54%). This phenomenon was ascribed to the decomposition of ATPEPDPA-produced polyphosphoric acid and poly(metaphosphoric acid) at relatively low temperatures, which dehydrated cellobiose to char and significantly reduced the quantity of levoglucose converted to combustible gases.

Under air atmosphere, the ATPEPDPA-treated cotton exhibited a thermal decomposition profile similar to that of the nitrogen atmosphere. After the first weight loss inflection point, a second weight loss peak was observed for both before and after cotton treatments (Figure 8d), and the weight loss for control cotton was 0 at 700 °C and that of ATPEPDPA-treated cotton decreased to 14.23%. This phenomenon was because the aliphatic char produced by cotton fibers at higher temperatures (above 450 °C) was oxidized to produce more stable aromatic char [39]. The treated cotton had a lower crest at this phase, which might have been caused by the fact that ATPEPDPA was a phosphorus-based flame retardant. Moreover, the phosphoric acid produced by the phosphorus facilitated the conversion of cotton fibers into an oxygen barrier char layer, further reducing the oxidation of the cotton matrix.

### 3.5. Combustion Characteristics

**Figure 9:**
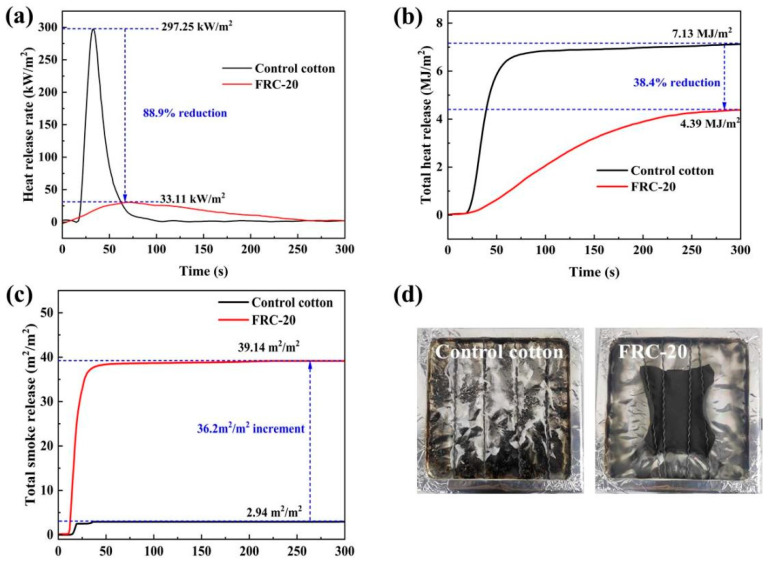
HRR (**a**), THR (**b**) and TSR (**c**) profiles of samples; (**d**) combustion pictures of samples during the cone calorimetric tests.

The heat release process of samples was analyzed using the cone calorimetric tests, and results are plotted in Figure 9 and Table 4. The heat release rate (HRR) of the control cotton increased rapidly and reached a peak (PHRR) at 33 s. The HRR curve slope of FRC-20 was smaller in comparison to that of the control cotton, and its heat release process was slow, with a 38.4% and 88.9% decrease in total heat release (THR) (4.39 MJ/m^2^ vs. 7.13 MJ/m^2^) and PHRR (33.11 kW/m^2^ vs. 297.25 kW/m^2^), respectively. A small amount of soft off-white ash remained after the combustion of the control cotton (Figure 9d). The FRC-20 became a black char layer after the test. This phenomenon indicated that the ATPEPDPA facilitated the dehydration of cotton fibers into char during combustion, thus decreasing the quantity for flammable gases produced by the pyrolysis of the cotton matrix at high temperatures. Thus, the heat released during the burning of the cotton matrix was significantly decreased. The more char the cotton matrix produced during combustion, the better the fire resistance of the treated cotton, meaning less heat was released from the treated cotton [40]. The fire growth rate (FGR) is an indicator of assessing the rapidity of fire expansion [41]. The higher the value of FGR, the faster the rate of burning of the material. The FGR value for FRC-20 was obviously smaller as compared to the control cotton (0.42 (kW/m^2^)/s vs. 9.01 (kW/m^2^)/s). The THR, HRR and FGR results showed that the ATPEPDPA-treated cotton in a real fire had little heat release and that ATPEPDPA delayed PHRR (78 s vs. 33 s), which would gain time for moving injured people and property.

After cotton fibers were heated, the FRC-20 residues were higher than control cotton. Moreover, CO_2_/CO, an indicator for evaluating the complete combustion of the cotton, was much lower in the FRC-20, which was in accordance with the THR data. Furthermore, the total smoke release (TSR) value of the FRC-20 was much higher than that of the control cotton (39.14 m^2^/m^2^ vs. 2.94 m^2^/m^2^). This result showed that ATPEPDPA reduced the production of flammable gas in the cotton fabric during the burning process, which caused incomplete combustion of the cotton fabric. This destroyed the flaming chain reaction, finally making the cotton fabric form a char layer, thereby reflecting the efficient flame retardancy of ATPEPDPA.

### 3.6. TG-FTIR Analyses

**Figure 10:**
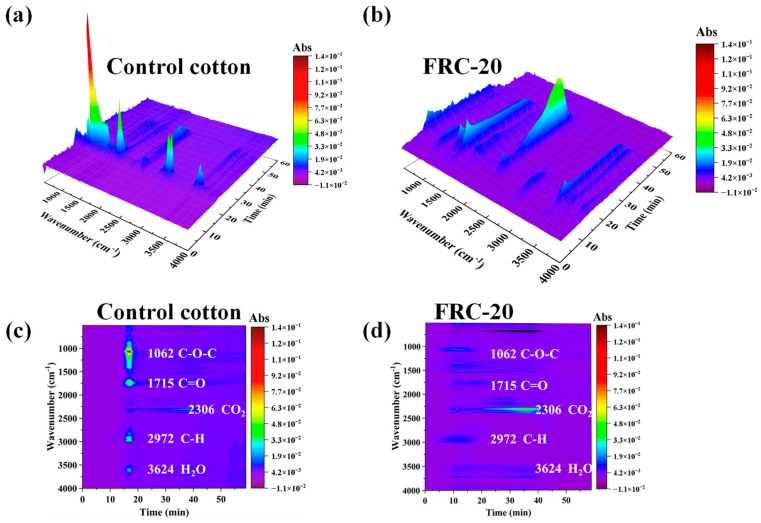
3D (**a**,**b**) and 2D (**c**,**d**) TG-FTIR pictures of control cotton and FRC-20.

Figure 10 shows the amount of volatile gas of the samples during the heating process. As shown in the three-dimensional plot, the gases from control cotton peaked at 16 min. From the two-dimensional projection diagram, these gas products peaked at 1062, 1715, 2306, 2972 and 3624 cm^−1^ for ether, carbonyl, carbon dioxide, hydrocarbon and water, respectively [42]. The FRC-20 displayed an earlier signal of gaseous products and a lower peak of pyrolysis products than the control cotton, and this was in accordance with the TG results. This observation suggested that dehydration and decomposition started at a lower temperature when the treated cotton was exposed to thermal radiation and that the quantity of flammable volatiles (ethers, carbonyls and hydrocarbons) and the production of refractory volatiles (water, carbon dioxide) decreased and increased, respectively, in the treated cotton due to the ATPEPDPA treatment. 

**Figure 11:**
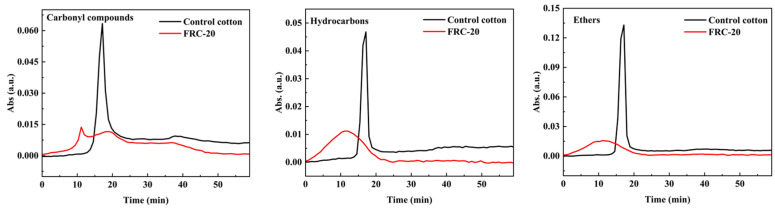
The absorption intensity curves of three representative pyrolysis volatiles.

Figure 11 compares the amounts of three volatiles before and after ATPEPDPA treatment. When contrasting the two curves, the crest strengths of carbonyl compounds, hydrocarbons and ethers of the FRC-20 were relatively smaller compared to the control cotton (0.014 vs. 0.064; 0.011 vs. 0.047; 0.016 vs. 0.133). This result, combined with TG and the cone calorimetric test results, suggested that ATPEPDPA decomposed at a lower temperature to produce phosphorus-containing acids when the treated cotton ignited. Cotton fibers in an acidic environment were converted toward the path of dehydration into char, and only a small amount of fibers degraded into flammable gases (including carbonyl compounds, hydrocarbons and ethers), suggesting that ATPEPDPA altered the pyrolysis process of cotton fabrics and interfered with the chain cycle reaction of combustion, thus endowing cotton fabrics with efficient flame retardancy.

### 3.7. Analysis of Char Residues

**Figure 12:**
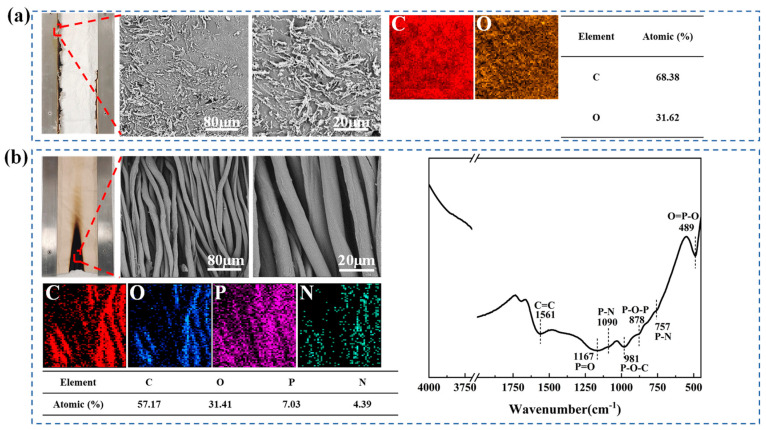
SEM images, EDS and FT-IR of residues: SEM and EDS of the control cotton residues (**a**); SEM, EDS and FT-IR of the FRC-20 residues (**b**).

Figure 12 shows the SEM, EDS and FT-IR profiles of the samples after heating. The fiber bundles were completely broken after the control cotton was heated, leaving only some ashes. The micro-region of FRC-20’s fiber bunches was maintained; its fibers were closely arranged, but its elemental content changed. The elemental content of carbon, phosphorus and nitrogen increased to 57.17, 7.03 and 4.39%, respectively, whereas that of oxygen decreased to 31.41%. In the FT-IR of the FRC-20 residues, C=C at 1561 cm^−1^ indicated that a stable aromatic carbon was formed after combustion [43]. The peaks at 1167, 1090, 981, 757 and 489 cm^−1^ represented the characteristic structure of ATPEPDPA [44,45,46]. A P-O-P absorption crest at 878 cm^−1^ suggested that the decomposition of ATPEPDPA during the heating process produced phosphoric acid and poly(metaphosphoric) acid, providing an acidic environment for cotton [47]. Overall, ATPEPDPA decomposed in the cotton fabric to produce phosphorus-containing acid, dehydrating the surface fibers and transforming into a char layer. Moreover, ATPEPDPA decomposition prevented the fiber matrix from contacting oxygen and other flammable components, demonstrating the condensed flame retardancy mechanism of ATPEPDPA.

### 3.8. Analysis of Physical Properties

Table 5 shows three physical properties’ test data of the samples. The whiteness of the ATPEPDPA-treated cotton decreased as the weight gain for samples increased. The whiteness of the FRC-20 dropped from 90.1 to 80.0%, due to the partial oxidation of cotton fibers when exposed to high temperatures [48]. The tensile strength of the ATPEPDPA-treated cotton showed similar whiteness trends, decreasing from 1845 and 1613 N in warp and weft directions, respectively, to 1441 and 1294 N in FRC-10 and finally to 1273 and 1176 N in FRC-20. The bending lengths of FRC-10, FRC-15 and FRC-20 increased with increasing concentrations of ATPEPDPA. Compared to the control cotton, the bending lengths of FRC-20 in different directions were 62.5 and 52.7 mm, only increasing by 17.9 and 9.8%, respectively. This phenomenon suggested that ATPEPDPA had a weak influence on the softness of cotton fibers, and the ATPEPDPA-treated cotton maintained a good hand feel.

## 4. Conclusions

In this work, an efficient and durable flame retardant, ATPEPDPA, containing -N-P(=O)-(O-CH_3_)_2_ and -N-P(=O)-(ONH_4_)_2_ groups was successfully synthesized and grafted to cotton textiles. The treated cotton had relatively high fire resistance and met the AATCC 61-2013 3A standard after 50 washings, suggesting high durability. The ATPEPDPA flame retardant exhibited the condensed flame retardance mechanism. The physical properties of the cotton after treatment were well maintained. The introduction of the N-P(=O)-(O-CH_3_)_2_ group reduced the tendency of the phosphoric acid group to combine with ions during the washing process, thereby enhancing fire resistance. The results showed that introducing N-P(=O)-(O-CH_3_)_2_ and -N-P(=O)-(ONH_4_)_2_ groups into ATPEPDPA was an efficient method to improve the durability of cotton’s fire retardance because the p-π conjunction between the N atom and P=O group decreased the polarization for -N-P(=O)-O-C, making it stable.

## Figures and Tables

**Table 1:** The weight gain (%) of samples.

Samples	FRC-10	FRC-15	FRC-20
FRC (wt%)	10	15	20
Weight gain (%)	11.5 ± 0.2	16.0 ± 0.5	18.6 ± 0.3

**Table 2:** The weight gain and VFT values of samples.

Samples	Weight Gain (%)	After-Flame Time (s)	After-Glow Time (s)	Char Length (cm)
Control cotton	-	41	43	0
FRC-10	11.5 ± 0.2	0	0	5.7 ± 0.2
FRC-15	16.0 ± 0.5	0	0	5.1 ± 0.1
FRC-20	18.6 ± 0.3	0	0	4.8 ± 0.2
FRC-10 after 50 LCs	8.0 ± 0.2	0	0	7.0 ± 0.3
FRC-15 after 50 LCs	11.3 ± 0.4	0	0	6.5 ± 0.1
FRC-20 after 50 LCs	14.0 ± 0.2	0	0	5.2 ± 0.2

**Table 3:** Comparative durability data of samples.

	Samples	WG (%)	LOI (%)	P Atomic Percentage (%)	Durability	LOI (%) after 50 LCs	P % after 50 LCs
Author	FRC-20	18.6	48.4	2.67	AATCC 61-2013 3A	44.6	2.46
(Jia et al., 2017) [36]	APTTP-treated cotton	22.4	43.8	-	AATCC 61-2006	26.9	-
(Ding et al., 2022) [37]	AATMPEG-treated cotton	17.1	45.0	3.52	AATCC 61-2013 2A	33.0	3.14
(Zhao et al., 2022) [18]	DETA-P-treated cotton	20.1	40.7	2.84	AATCC 61-2013 2A	29.0	2.13
(Lu et al., 2022) [38]	ASTP-treated cotton	33.1	45.2	2.58	AATCC 61-2013 2A	32.1	2.30

**Table 4:** Quantitative data of samples at the cone calorimetric tests.

Samples	PHRR (kW/m^2^)	T_PHRR_ (s)	FGR ((kW/m^2^)/s)	THR (MJ/m^2^)	CO_2_/CO	TSR (m^2^/m^2^)	Residue (%)
Control cotton	297.25	33	9.01	7.13	101.62	2.94	5.0
FRC-20	33.11	78	0.42	4.39	4.52	39.14	18.3

**Table 5:** The physical properties’ data of samples.

Samples	Whiteness	Tensile Strength (N)		Bending Length (mm)	
		Warp	Weft	Warp	Weft
Control cotton	90.1	1845 ± 10	1613 ± 7	53.0 ± 1.8	48.0 ± 1.9
FRC-10	82.2	1441 ± 12	1294 ± 21	59.8 ± 0.9	49.7 ± 3.3
FRC-15	80.2	1374 ± 9	1215 ± 15	61.2 ± 2.6	51.9 ± 0.7
FRC-20	80.0	1273 ± 11	1176 ± 13	62.5 ± 1.2	52.7 ± 1.6

## Data Availability

Data are contained within the article.
